# Exploiting the exceptional biosynthetic potency of the endophytic *Aspergillus terreus* in enhancing production of Co_3_O_4_, CuO, Fe_3_O_4_, NiO, and ZnO nanoparticles using bioprocess optimization and gamma irradiation

**DOI:** 10.1016/j.sjbs.2021.12.019

**Published:** 2021-12-13

**Authors:** El-Sayed R. El-Sayed, Shaimaa A. Mousa, Dalia A.M. Abdou, Mohamed A. Abo El-Seoud, Adel A. Elmehlawy, Samar S. Mohamed

**Affiliations:** a Plant Research Department, Nuclear Research Center, Egyptian Atomic Energy Authority, Cairo, Egypt; b Microbiology Department, Faculty of Science, Ain Shams University, Cairo, Egypt

**Keywords:** Nanoparticles, *Aspergillus terreus*, Response surface methodology, Optimization, Gamma irradiation, Endophytic

## Abstract

Developing a suitable applicative process and scaling up the microbial synthesis of nanomaterials is an attractive and emerging prospect for a future sustainable industrial production. In this paper, optimization of fermentation conditions for enhanced production of Co_3_O_4_, CuO, Fe_3_O_4_, NiO, and ZnO nanoparticles by the endophytic *A. terreus* ORG-1 was studied. Different cultivation conditions were evaluated. Then, a response surface methodology program was used to optimize physical conditions controlling the biosynthesis of these NPs. Finally, the use of gamma irradiation for improvement of NPs’ production was adopted. Under the optimum conditions and after gamma irradiation, the final yields of the respective NPs reached 545.71, 651.67, 463.19, 954.88, 1356.42 mg L^−1^. To the best of our knowledge, this is the first report on the production and enhancement of different types of nanomaterials from one microbial culture that can open up the way towards the industrialization of the microbial production of nanomaterials.

## 1 Introduction

Recently, several studies in bio-nanotechnology have mainly focused on the microbial synthesis of different nanomaterials with myriad applications. Despite that, newer areas of investigation have emerged including the exploitation of microorganisms for developing a sustainable production of these nanomaterials (Lateef et al., 2021). Traditionally, the synthesis of nanomaterials is performed using physical- or chemical-based methodologies. Unfortunately, these methods had negative influences on human health and ecosystems due to the resultant hazardous toxic wastes. Besides, such methods had high economic costs and were very complicated. Accordingly, a pressing scientific and social need has emerged in order to overcome these shortcomings. In this regard, exploring newer, alternative, and green approaches to develp a sustainable process for synthesis of nanomaterials becomes an essential target (Ljaz et al., 2017). Most recently, several studies suggested that microorganisms could be promising alternatives to develop a facile, rapid, and eco-friendly platforms for production of several types of nanomateroials (Lateef et al., 2021, and references therein). Such platforms proved to be powerful tools for prospective large-scale production with an excellent ability to improve and modify (Dorcheh and Vahabi, 2016). Fungal platforms, particularly endophytes, have magnificently emerged as economic biological factories of nanomaterials (Abdelhakim et al., 2020) due to their tolerance, flexibility, easy maintenance, flexibility, and tolerance (Rai et al., 2021). Further more, endophytic fungi had enhanced metabolic activities more than their free counterparts (El-Sayed et al., 2020d), so they are potential, reliable, excellent sources of a huge number of biologically active metabolites (El-Sayed, 2021) with several applications. Thus, endophytic fungi may be a key role in the replacement of the chemical and physical methods.

We have already described the synthesis of Co_3_O_4_, CuO, Fe_3_O_4_, NiO, and ZnO (nanoparticles) NPs using the endophytic *Aspergillus terreus* (Mousa et al., 2021). Due to their unique properties when compared to their metals, these NPs have magnificently emerged in several applications. Certainly, they proved to be ideal candidates for several medical, industrial, and agricultural applications including batteries, gas sensors, energy storage systems, and human healthcare products (Co_3_O_4_NPs, Omran et al., 2020), electronic, optical, and cytotoxic activities (CuONPs, El-Batal et al., 2020), cancer therapy, drug delivery, wastewater treatment, and antimicrobial agents (Fe_3_O_4_NPs, Mahanty et al., 2019), superconductivity, adsorption of toxic pollutants, and anti-inflammation (NiONPs, Atalay et al., 2016), and catalytic properties, antibacterial, antioxidant, semiconducting, and anticancer activities (ZnONPs, Abdelhakim et al., 2020).

As a part of our continuing search in this regard, we aim in this paper to harness this exceptional potentiality of *Aspergillus terreus* as a bio-factory for the production of these NPs. Numerous factors could potentially affect the process of biosynthesis of NPs. In the literature, different environmental factors such as culture filtrate, temperature, mixing time, precursor concentration, and pH were extensively investigated to optimize and maximize the production of NPs. In addition, reaction conditions such as metal ions concentration, contact time, and culture filtrate usually play a vital role in increasing the production rate NPs (Mohamed et al., 2019). In the same connection, gamma-irradiation can significantly enhance the NPs manufacturing by increasing the active metabolites responsible for the formation of NPs. In this paper, optimization of fermentation conditions for enhanced production of the five types of NPs by this strain was studied. Furthermore, response surface methodology optimization of reaction conditions controlling the formation of these NPs was also investigated. Finally, the use of gamma irradiation for the improvement of NPs’ production by the fungal strain was adopted.

## 2 Materials and methods

### 2.1 Fungal strain

The endophytic fungus *Aspergillus terreus* ORG-1 was used for the preparation of nanoparticles. The strain was isolated (from the leaves of *Origanum majorana*) and identified according to our previous study (Mousa et al., 2021).

### 2.2 Inoculum preparation and synthesis of Co_3_O_4_, CuO, Fe_3_O_4_, NiO, and ZnO nanoparticles

Fungal culture (7-days old) of *Aspergillus terreus* ORG-1 was harvested and used to make spore suspension with a final concentration adjusted to 10^6^ spore mL^−1^. 250 mL Erlenmeyer flasks containing 50 mL PD broth were prepared, adjusted to pH 6.0, sterilized, and cooled to room temperature. After which, 1 mL of this suspension was added aseptically to each flask. These flasks were incubated under static conditions at 30 °C for 7 days then filtered through Whatman No.1 filter papers and the obtained cell-free filtrate was used for the preparation of NPs.

Synthesis of Co_3_O_4_, CuO, Fe_3_O_4_, NiO, and ZnO NPs was carried out according to our previous study (Mousa et al., 2021). In brief, five different aqueous solutions with a concentration of 10 mM of ZnC_4_H_6_O_4_·7H_2_O, CuSO_4_·5H_2_O, NiSO_4_·6H_2_O, Fe(NO_3_)_3_·9H_2_O, and CoSO_4_·7H_2_O (all salts were purchased from Sigma-Aldrich, St Louis, MO, USA) were prepared. The obtained cell-free filtrate was then mixed with an equal volume of each aqueous solution separately. Finally, the five reaction mixtures were maintained for 2 h under vigorous stirring at room temperature.

### 2.3 Separation, purification, and yield estimation of NPs

The obtained reaction mixtures were taken to separate and purify the synthesized NPs. Ultra-centrifugation at 20,000 rpm (20 min) was applied to collect the synthesized NPs. The collected NPs were then washed (in deionized water subsequently in ethanol) and finally dried at 50 °C. The obtained five different powders of NPs were treated ultrasonically after dissolving individually in HPLC-grade ethanol, to disperse the individual NPs.

The concentration of the synthesized NPs was measured after recording the absorption at 230 nm for Co_3_O_4_NPs, 256 nm for CuONPs, 285 nm for Fe_3_O_4_NPs, 330 nm for NiONPs, and 370 nm for ZnONPs, against standard curves for each type of NPs (Mousa et al., 2021). The yield was estimated using a standard curve for each type of NPs and expressed as mg NPs L^−1^ cell-free filtrate.

### 2.4 Estimation of fungal growth

The biomass yield (mycelim and spores) was estimated after cultivation by filtering the culture broth through pre-weighed filter papers (Whatman No 1). Before estimating the dry biomass, the collected cells were dried at 60 °C to a constant weight.

### 2.5 Experimental design

The effect of several fermentation conditions on production of NPs by the fungal strain were studied by OFAT (One factor at a time) approach. Then, response surface methodology (RSM) was used to study the physical conditions.

#### 2.5.1 Effect of fermentation conditions

Different fermentation conditions that were suggested to affect the biosynthesis of Co_3_O_4_NPs, CuONPs, Fe_3_O_4_NPs, NiONPs, and ZnONPs by the ORG-1 strain were studied. These conditions were six different fermentation broth media (Table 1), different incubation periods by incubating the flasks for 15 days, incubation temperatures, initial pH-values of the fermentation medium (adjusted using 1 N HCl or 1 N NaOH), medium volume, inoculation sizes, and inoculum ages.

**Table 1:** Effect of different broth media on growth (g L^−1^) and production of Co_3_O_4_NPs, CuONPs, Fe_3_O_4_NPs, NiONPs, and ZnONPs (mg L^−1^) by *A. terreus* ORG-1.

Broth medium	Dry biomass (g L^−1^)	NPs concentration (mg L^−1^)				
		Co_3_O_4_NPs	CuONPs	Fe_3_O_4_NPs	NiONPs	ZnONPs
1. PD (C)	7.24 ± 0.12^d^	7.86 ± 0.21^c^	8.51 ± 0.21^e^	2.76 ± 0.17^c^	1.98 ± 0.31^d^	25.14 ± 0.12^e^
2. CDYE	14.63 ± 0.34^a^	8.74 ± 0.87^bc^	17.90 ± 0.34^c^	3.89 ± 0.52^b^	2.05 ± 0.11^d^	35.76 ± 0.06^d^
3. CD	9.04 ± 0.18 ^cd^	4.53 ± 0.34^d^	20.07 ± 0.87^b^	1.87 ± 0.88^d^	1.76 ± 0.04^d^	23.34 ± 0.36^e^
4. SAB	10.02 ± 0.41^c^	11.53 ± 0.91^a^	25.53 ± 0.91^a^	5.95 ± 0.69^a^	15.85 ± 0.76^a^	60.73 ± 0.54^a^
5. YES	12.52 ± 0.54^b^	10.53 ± 0.21^ab^	20.21 ± 0.56^b^	5.91 ± 0.24^a^	9.41 ± 0.34^b^	52.13 ± 0.14^b^
6. MEA	13.73 ± 0.65^ab^	7.65 ± 0.82^c^	12.87 ± 0.73^d^	4.95 ± 0.55^ab^	4.03 ± 0.12^c^	43.08 ± 0.22^c^

1. Potato dextrose (g L^−1^): potato infusion 200, D-glucose 20.

2. Czapek-Dox’s supplemented with 0.5 % yeast extract (g L^−1^): sucrose 30, yeast extract 5, KH_2_PO_4_ 0.5, KCl 0.5, MgSO_4_·7H_2_O 0.5, FeSO_4_·7H_2_O 0.01.

3. Czapek-Dox’s (g L^−1^): sucrose 30, NaNO_3_ 3, KH_2_PO_4_ 0.5, KCl 0.5, MgSO_4_·7H_2_O 0.5, FeSO_4_·7H_2_O 0.01.

4. Sabouraud’s-glucose (g L^−1^): peptone 10, glucose 20, MgSO_4_·7H_2_O 1.0, KH_2_PO_4_ 1.0.

5. Yeast-sucrose (g L^−1^): sucrose 50, yeast extract 20.

6. Malt extract autolysate (g L^−1^): Malt extract 30, peptone 1, glucose 2, CuSO_4_·5H_2_O 0.005, ZnSO_4_·7H_2_O 0.01.

Initial pH of all tried media was adjusted to 6.0 using 1 N NaOH and HCl. Static cultures were carried out for 7 days at 30 °C using an inoculum size of 1 mL/50 mL medium. Calculated mean is for triplicate measurements from two independent experiments ± SD, ^a-e^ means with different superscripts in the same column are considered statistically different (LSD test, *P* ≤ 0.05).

#### 2.5.2 Optimization of physical conditions

Response surface methodology (RSM) program was used to study the optimum levels of physical conditions controlling the biosynthesis of Co_3_O_4_NPs, CuONPs, NiONPs, Fe_3_O_4_NPs, and ZnONPs by the cell-free filtrate of the ORG-1 strain. These parameters were reaction time (min), the volume of the cell-free filtrate (L), and salt concentration (mM). A three-level Box–Behnken (BB) design (Design-Expert software version 8.0.7.1) was used to optimize these parameters and to analyze their relationships (Table 2), and the details of the trails for the studied parameters are listed in Table 3. The five types of NPs were synthesized under the optimum conditions from the OFAT approach. Finally, the yield of NPs was then estimated, as previously mentioned.

**Table 2:** Reaction conditions controlling biosynthesis of NPs and their levels used in BB design.

Factor	Symbol	Level		
		−1	0	1
Reaction time (min)	A	60	240	420
Volume of culture filtrate (L)	B	1	2	3
Salt concentration (mM)	C	5	10	15

**Table 3:** BB experimental design matrix representing the response of NPs production (mg L^−1^ culture filtrate) by *A. terreus* ORG-1.

Run	Factor			Response: NPs concentration (mg L^−1^ culture filtrate)									
				Co_3_O_4_NPs		CuONPs		Fe_3_O_4_NPs		NiONPs		ZnONPs	
	A	B	C	Actual	Predicted	Actual	Predicted	Actual	Predicted	Actual	Predicted	Actual	Predicted
1	−1	−1	0	108.31 ± 1.11	107.46	27.63 ± 1.09	22.56	80.71 ± 1.11	78.91	252.12 ± 3.14	227.86	248.52 ± 2.78	238.20
2	1	−1	0	119.87 ± 3.89	114.70	115.87 ± 2.01	111.13	103.59 ± 1.54	105.24	408.54 ± 2.87	384.79	438.91 ± 5.19	414.78
3	−1	1	0	147.88 ± 1.46	153.05	37.51 ± 4.12	42.25	107.27 ± 1.54	105.62	450.19 ± 5.91	473.94	217.66 ± 2.44	241.79
4	1	1	0	238.99 ± 5.31	239.84	225.99 ± 2.87	231.06	156.06 ± 2.19	157.86	578.38 ± 2.77	602.64	357.65 ± 3.91	367.97
5	−1	0	−1	108.76 ± 1.27	103.35	15.77 ± 0.76	14.98	58.77 ± 1.44	60.38	157.66 ± 3.18	133.32	166.92 ± 2.44	151.68
6	1	0	−1	134.87 ± 2.87	133.78	137.87 ± 4.89	136.75	83.76 ± 2.91	81.92	145.72 ± 1.33	120.87	241.65 ± 1.67	240.22
7	−1	0	1	124.45 ± 2.18	125.54	64.66 ± 1.32	65.78	140.28 ± 1.26	142.12	202.02 ± 2.87	226.87	287.52 ± 3.88	288.95
8	1	0	1	183.76 ± 1.11	189.16	220.61 ± 3.14	221.39	200.77 ± 2.78	199.16	500.62 ± 6.22	524.96	487.91 ± 2.61	503.15
9	0	−1	−1	95.44 ± 1.05	101.70	70.98 ± 1.78	76.84	11.87 ± 0.56	12.06	190.74 ± 1.67	239.34	320.04 ± 2.78	345.59
10	0	1	−1	136.87 ± 2.44	137.11	53.89 ± 1.88	49.94	45.31 ± 0.71	45.35	299.77 ± 3.73	300.36	117.91 ± 1.32	109.02
11	0	−1	1	90.77 ± 1.01	90.53	43.89 ± 2.11	47.85	105.21 ± 3.98	105.17	317.81 ± 2.14	317.22	321.84 ± 5.13	330.73
12	0	1	1	232.11 ± 1.45	225.85	220.23 ± 2.11	214.37	151.39 ± 4.29	151.20	768.71 ± 8.43	720.11	549.64 ± 6.98	524.08
13	0	0	0	410.65 ± 5.32	415.86	318.64 ± 4.12	323.78	215.54 ± 2.55	214.33	525.32 ± 1.44	565.51	864.54 ± 7.32	849.19
14	0	0	0	415.87 ± 2.11	415.86	327.81 ± 3.19	323.78	213.39 ± 3.14	214.33	627.01 ± 2.67	565.51	852.67 ± 6.89	849.19
15	0	0	0	418.91 ± 3.56	415.86	326.54 ± 1.56	323.78	214.76 ± 2.17	214.33	524.82 ± 3.09	565.51	843.88 ± 4.05	849.19
16	0	0	0	416.21 ± 2.37	415.86	320.38 ± 3.65	323.78	215.29 ± 2.99	214.33	624.98 ± 2.88	565.51	855.43 ± 8.62	849.19
17	0	0	0	417.64 ± 3.17	415.86	325.55 ± 6.32	323.78	212.66 ± 2.81	214.33	525.43 ± 3.76	565.51	829.45 ± 4.22	849.194

SAB broth (pH 6.0) was used for cultivation. Static cultures were carried out for 9 days at 30 °C using an inoculum size of 2 mL/50 mL medium. Calculated mean is for triplicate measurements from two independent experiments.

The experimental data on the recorded concentration of Co_3_O_4_NPs, CuONPs, Fe_3_O_4_NPs, NiONPs, and ZnONPs were analyzed separately by analysis of variance test (ANOVA). The following equation illustrates the obtained behavior of the BB design:

*Y = β0 + Σβixi + Σβijxixj + Σβiixi*^2^

where *Y*; predicted response, *βi*; linear offset, *βo*; offset term, *βii*; squared offset, *βij*; interaction effect, *xi,* and *xj*; dependent coded variables. Statistical significance concerning the constructed model was estimated by Fisher’s F–test and the proportion of variance was given by R^2^ value, the multiple coefficients of determination. 3D response surface and 2D contour graphs were constructed based on the analysis of the software to study the interaction between variables.

Model validation Additional independent experiments were performed to validate the performance of the suggested optimum levels from the BB designs on maximizing the production of NPs. The actual concentration of the synthesized NPs and the model predicted yields were then compared.

### 2.6 Effect of ^60^Co gamma-irradiation

7–day old cultures of the *A. terreus* ORG-1 were washed by a buffer phosphate (pH 7.0). Spores were collected by centrifugation and then suspended in sterile distilled water. The freshly prepared spore suspension was irradiated by gamma rays at different doses of 0.25, 0.50, 1.00, 2.00, and 4.00 KGy (^60^Co Gamma chamber, MC20, Russia). After irradiation, spore suspensions were immediately kept in darkness at 4 °C overnight to avoid photoreactivation. Finally, 2 mL of the irradiated spore suspensions for every irradiation dose were added separately to 250 mL flasks containing 50 mL of SAB broth (pH 6.0) and incubated at 30 °C for 9 days, the optimum conditions from the OFAT approach. The five types of NPs were synthesized under the optimum conditions from the RSM approach. Finally, the yield of NPs was then estimated, as previously mentioned.

### 2.7 Statistics

Analysis of variance (One-Way ANOVA) and Least Significant Difference (LSD) tests (at 0.05 level) were used to analyze the statistical significance by SPSS software (V. 22, IBM Corp).

## 3 Results

### 3.1 Selection of the most favorable fermentation conditions

Generally, results of testing different fermentation conditions indicated that production of the five types of NPs by *A. terreus* ORG-1 was greatly influenced by the studied condition where a significant increase (*P ≤ 0.05*) in the obtained yield of the five types of NPs at all treatments by comparing to control. Table 1 presented the obtained results of the selection of the most proper fermentation medium for maximum production of NPs. The obtained results clearly indicated that maximum concentrations of Co_3_O_4_NPs (11.53 ± 0.91 mg L^−1^), CuONPs (25.53 ± 0.91 mg L^−1^), Fe_3_O_4_NPs (5.95 ± 0.69 mg L^−1^), NiONPs (15.85 ± 0.76 mg L^−1^), and ZnONPs (60.73 ± 0.54 mg L^−1^) were achieved on SAB medium.

**Fig. 1:**
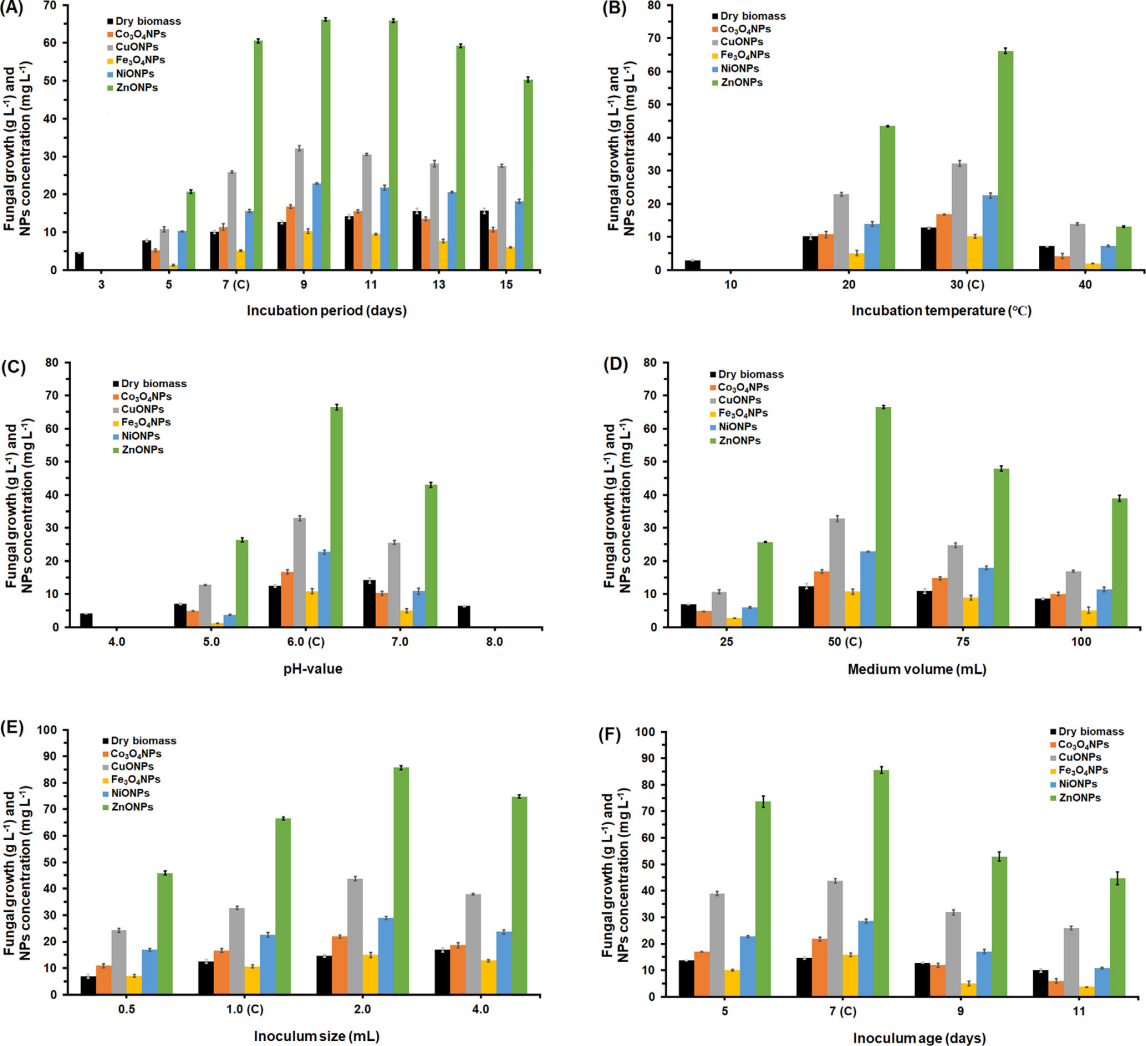
Effect of different incubation periods (A), incubation temperatures (B), pH-values (C), volumes of fermentation medium (D), inoculum sizes (E), and inoculum ages (F) on growth (g L^−1^) and production of Co_3_O_4_NPs, CuONPs, Fe_3_O_4_NPs, NiONPs, and ZnONPs (mg L^−1^) by *A. terreus* ORG-1. Data are shown as the mean ± SD of triplicate measurements from two independent experiments.

The time course profile of production of the five types of NPs production by the ORG-1 strain (Fig. 1A) showed that the production process started after 3 days of incubation. The obtained results also indicated that maximum yields of Co_3_O_4_NPs (16.81 ± 0.44 mg L^−1^), CuONPs (32.17 ± 0.71 mg L^−1^), Fe_3_O_4_NPs (10.25 ± 0.61 mg L^−1^), NiONPs (22.94 ± 0.21 mg L^−1^), and ZnONPs (66.21 ± 0.44 mg L^−1^) were attained after 9 days of incubation (Fig. 1A). Of all the tested incubation temperatures (Fig. 1B), 30 °C was the most proper for maximum production of the five types of NPs. The cell-free filtrate obtained from the fermentation conducted at 10 °C failed to reduce the metal salts of the five types of NPs, thereby no NPs were produced; however, mycelial growth was yet evident. Testing different initial pH-values showed that adjusting the initial pH-value of the SAB broth (the most proper one for maximum production rates) at 6 was the most proper for the production of NPs (Fig. 1C). In spite of the mycelial growth, the cell-free filtrate obtained at pH-values of 4 and failed to reduce the metal salts of the five types of NPs, thereby no NPs were produced. Results of testing different fermentation medium volumes (Fig. 1D), inoculum sizes (Fig. 1E), and inoculum ages (Fig. 1F) indicated that fermentation conducted using 2 mL inoculum size of the 7-day-old spore suspension for 50 mL medium promoted higher production rates for Co_3_O_4_NPs (21.75 ± 0.73 mg L^−1^), CuONPs (43.76 ± 0.83 mg L^−1^), Fe_3_O_4_NPs (15.88 ± 0.72 mg L^−1^), NiONPs (28.61 ± 0.76 mg L^−1^), and ZnONPs (85.61 ± 0.33 mg L^−1^).

### 3.2 Statistical optimization of reaction conditions

Parameters of reaction time (min), volume of fermentation medium (L), and salt concentration were subjected to RSM to detect their optimum levels and study their interactions. Table 3 presented the design matrices for the five modes used with the actual experimental, and predicted concentrations of the five types of NPs produced by the fungal strain. The recorded responses (NPs concentration) for each type of NPs were analyzed and the following equations were obtained:

*Co_3_O_4_NPs*_(mg L_^−1^_)_*= + 415.86* + *23.51A* + *42.68B* + *19.39C* + *19.89AB* + *8.30AC* + *24.98BC* − *131.47A*^2^ − *130.63B*^2^ − *146.43C*^2^

*CuONPs*_(mg L_^−1^_)_*= + 323.78* + *69.35A* + *34.91B* + *33.86C* + *25.06AB* + *8.46AC* + *48.36BC* − *104.78A*^2^ − *117.26B*^2^ − *109.28C*^2^

*Fe_3_O_4_NPs*_(mg L_^−1^_)_*= + 214.33* + *19.64A* + *19.83B* + *49.74C* + *6.48AB* + *8.88AC* + *3.19BC* − *29.99A*^2^ − *72.44B*^2^ − *63.45C*^2^

*NiONPs*_(mg L_^−1^_)_*= + 565.51* + *71.41A* + *115.98B* + *124.41C* − *7.06AB* + *77.63AC* + *85.47BC* − *142.98A*^2^ − *0.2260B*^2^ − *171.03C*^2^

*ZnONPs*_(mg L_^−1^_)_*= + 849.19* + *75.69A* − *10.81B* + *100.05C* − *12.60AB* + *31.42AC* + *107.48BC* − *282.43A*^2^ − *251.08B*^2^ − *270.76C*^2^

Each model was analyzed by ANOVA test (Table 4) and the obtained data indicated that the five models used for all the types of NPs are significant (“Prob > *F*” were lower than 0.05). Moreover, results presented in Table 4 showed that the model precisely represented the relationship between the three parameters controlling the production of NPs and the achieved concentration of NPs where the estimated coefficient of determination (R^2^) reached 0.9992 for Co_3_O_4_NPs, 0.9989 for CuONPs, 0.9995 for Fe_3_O_4_NPs, 0.9619 for NiONPs, and 0.9966 for ZnONPs (Table 4).

**Table 4:** Analysis of variance (ANOVA) for production of NPs by *A. terreus* ORG-1.

Source	Degree of freedom	Co_3_O_4_NPs		CuONPs		Fe_3_O_4_NPs		NiONPs		ZnONPs	
		F-Value	P-Value	F-Value	P-Value	F-Value	P-Value	F-Value	P-Value	F-Value	P-Value
Model	9	960.49	< 0.0001	710.36	< 0.0001	1896.66	< 0.0001	19.68	0.0004	231.35	< 0.0001
A	1	132.37	< 0.0001	1016.14	< 0.0001	714.89	< 0.0001	13.19	0.0084	79.90	< 0.0001
B	1	436.27	< 0.0001	257.46	< 0.0001	728.60	< 0.0001	34.80	0.0006	1.63	0.2426
C	1	90.07	< 0.0001	242.26	< 0.0001	4583.98	< 0.0001	40.04	0.0004	139.61	< 0.0001
A^2^	1	47.36	0.0002	66.35	< 0.0001	38.87	0.0004	0.0644	0.8069	1.11	0.3277
B^2^	1	8.25	0.0239	7.57	0.0285	72.96	< 0.0001	7.80	0.0268	6.88	0.0342
C^2^	1	74.70	< 0.0001	247.06	< 0.0001	9.40	0.0182	9.45	0.0180	80.56	< 0.0001
AB	1	2178.33	< 0.0001	1220.92	< 0.0001	876.70	< 0.0001	27.84	0.0012	585.56	< 0.0001
AC	1	2150.66	< 0.0001	1529.09	< 0.0001	5116.04	< 0.0001	0.0001	0.9936	462.75	< 0.0001
BC	1	2702.48	< 0.0001	1328.11	< 0.0001	3925.24	< 0.0001	39.83	0.0004	538.16	< 0.0001
Lack of Fit	3	6.51	0.0510	4.11	0.1030	5.12	0.0742	1.03	0.47	6.27	0.0542

Std. Dev.		5.78		6.15		2.078		55.61		23.95	
Mean		223.61		167.87		136.27		417.64		470.71	
C.V. %		2.58		3.67		1.53		13.32		5.088	
R^2^		0.9992		0.9989		0.9995		0.9619		0.9966	
Adjusted R^2^		0.9982		0.9975		0.9990		0.9131		0.9923	
Predicted R^2^		0.9890		0.9864		0.9946		0.7010		0.9549	
Adeq Precision		73.388		65.435		126.913		14.051		40.296	

**Fig. 2:**
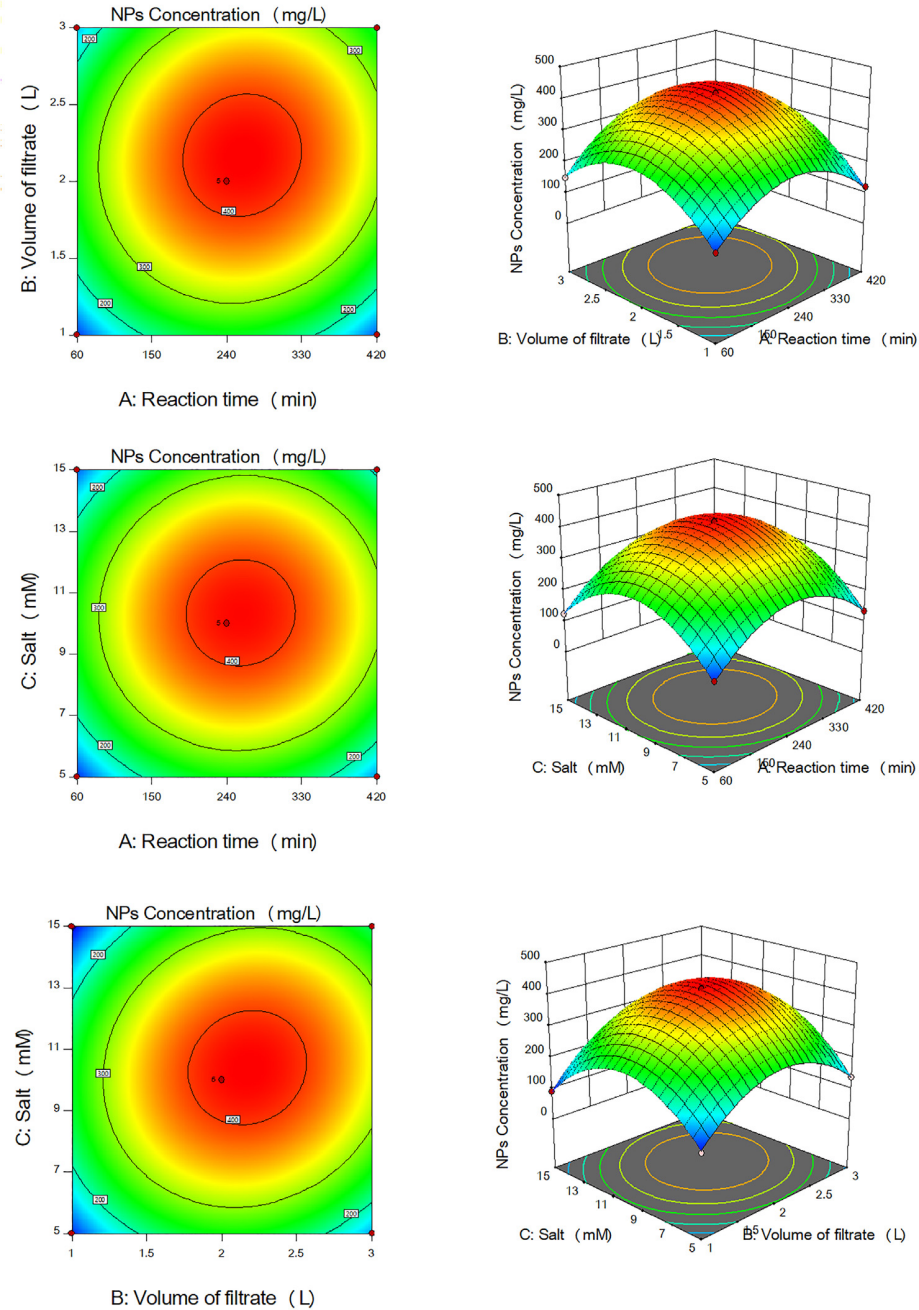
3D surface and 2D contour plots showing the effect of different factors on production of Co_3_O_4_NPs by *A. terreus* ORG-1. (a) Effect of reaction time and volume of the cell-free filtrate. (b) Effect of reaction time and salt concentration. (c) Effect of volume of the cell-free filtrate and salt concentration.

**Fig. 3:**
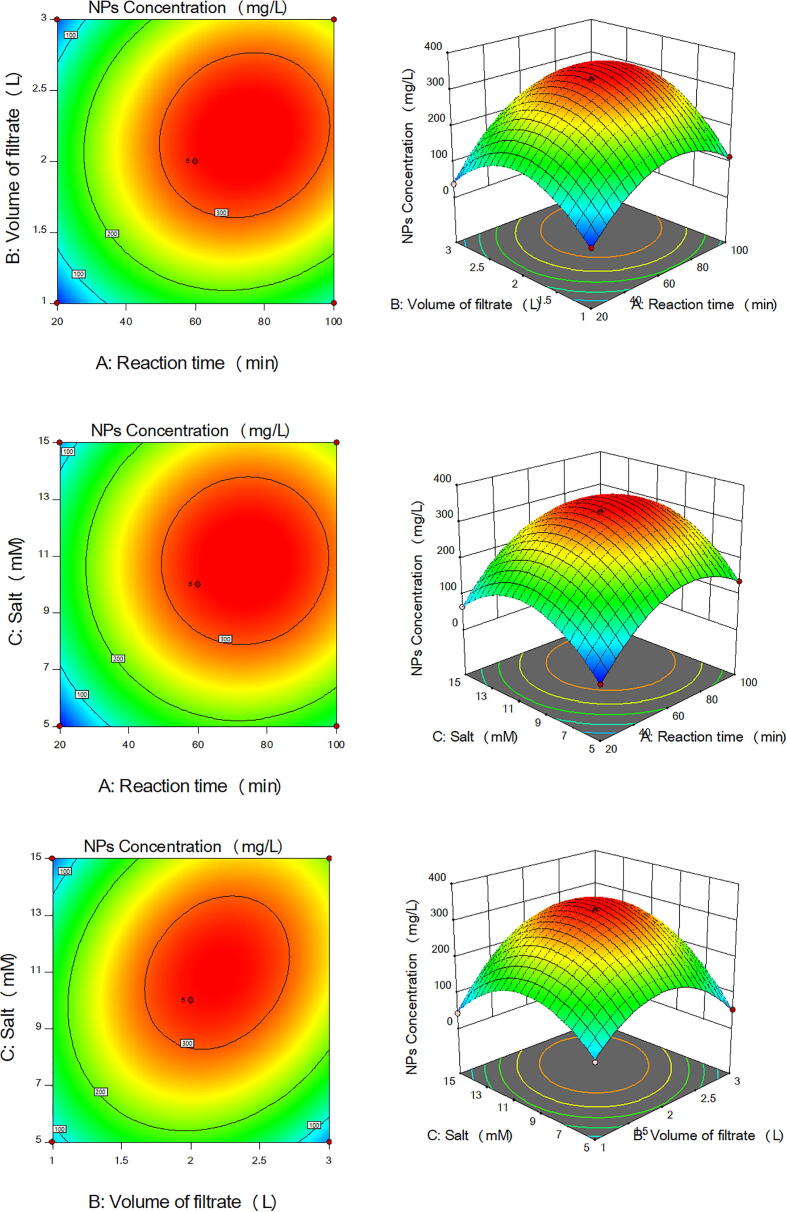
3D surface and 2D contour plots showing the effect of different factors on production of CuONPs by *A. terreus* ORG-1. (a) Effect of reaction time and volume of the cell-free filtrate. (b) Effect of reaction time and salt concentration. (c) Effect of volume of the cell-free filtrate and salt concentration.

**Fig. 4:**
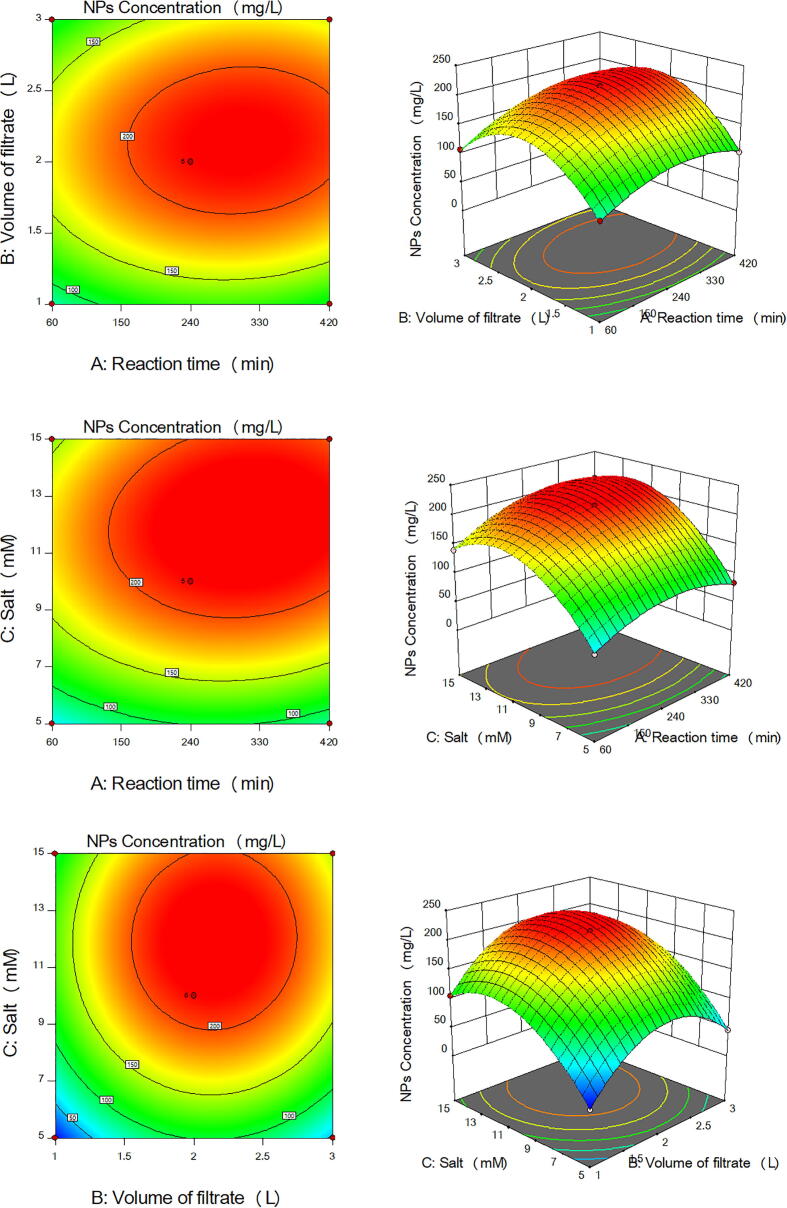
3D surface and 2D contour plots showing the effect of different factors on production of Fe_3_O_4_NPs by *A. terreus* ORG-1. (a) Effect of reaction time and volume of the cell-free filtrate. (b) Effect of reaction time and salt concentration. (c) Effect of volume of the cell-free filtrate and salt concentration.

**Fig. 5:**
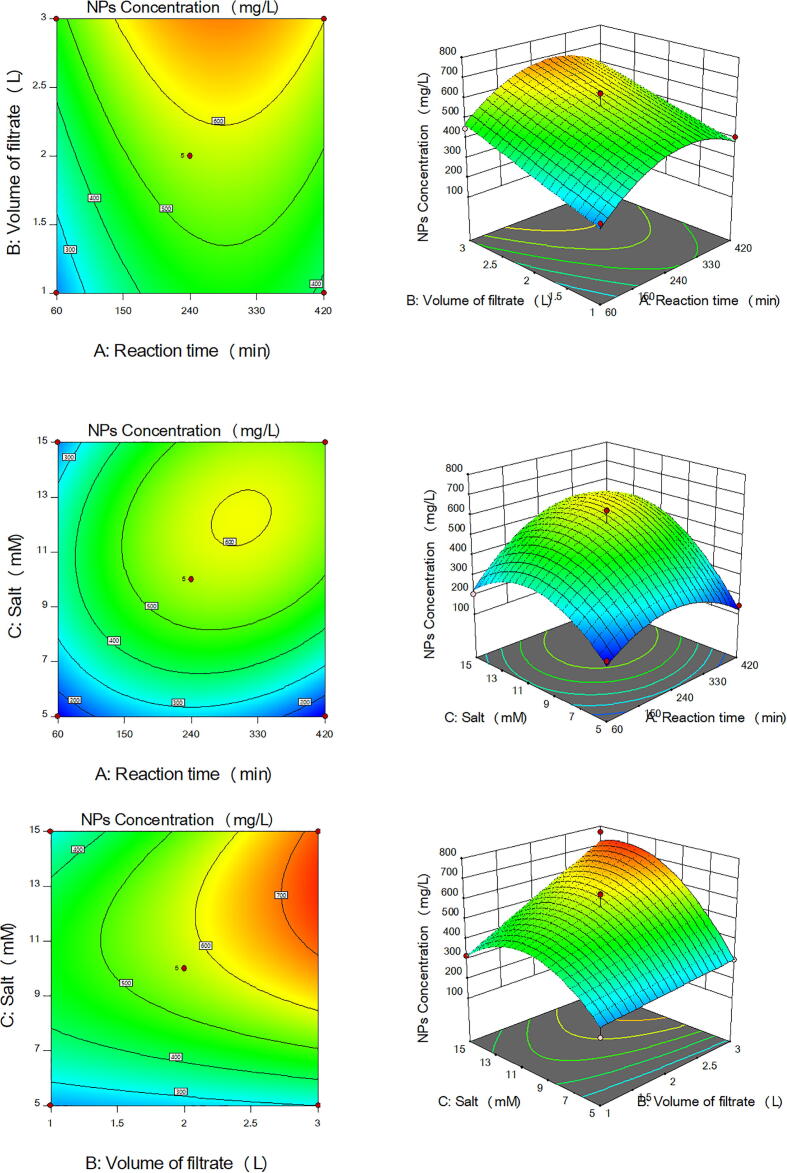
3D surface and 2D contour plots showing the effect of different factors on production of NiONPs by *A. terreus* ORG-1. (a) Effect of reaction time and volume of the cell-free filtrate. (b) Effect of reaction time and salt concentration. (c) Effect of volume of the cell-free filtrate and salt concentration.

**Fig. 6:**
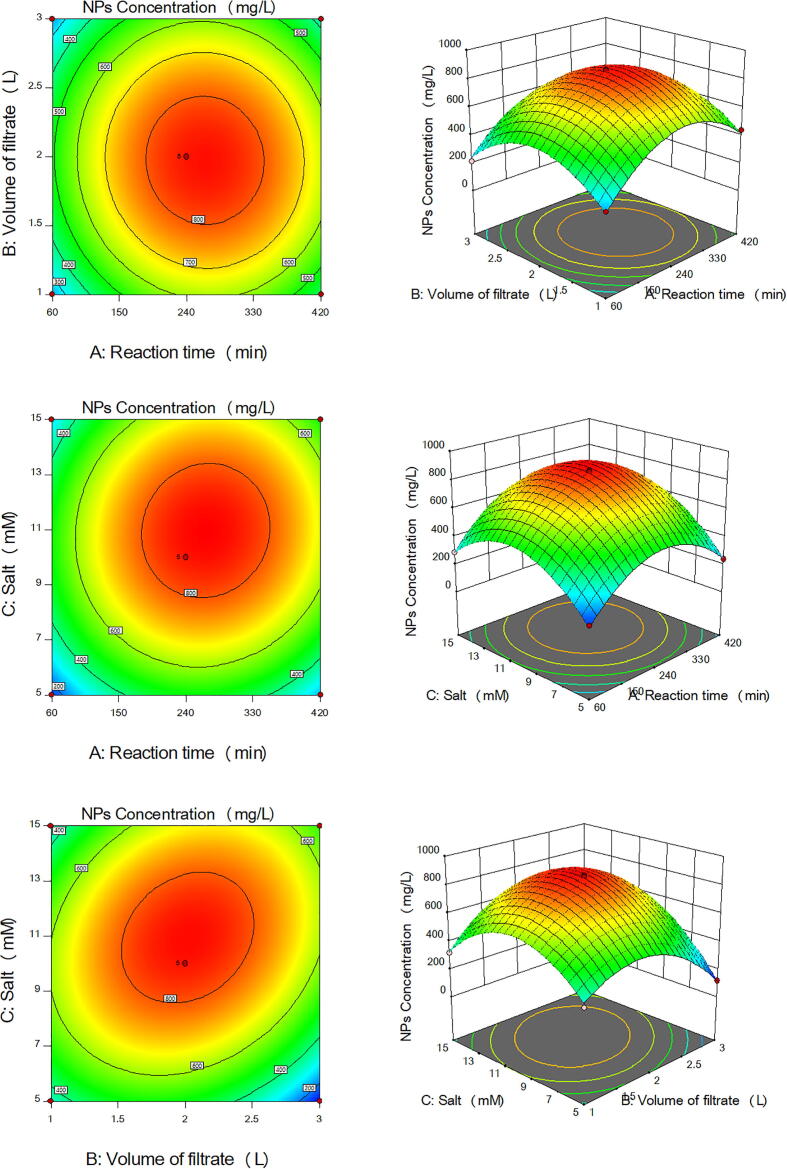
3D surface and 2D contour plots showing the effect of different factors on production of ZnONPs by *A. terreus* ORG-1. (a) Effect of reaction time and volume of the cell-free filtrate. (b) Effect of reaction time and salt concentration. (c) Effect of volume of the cell-free filtrate and salt concentration.

The main effects and the interaction between the three parameters controlling the production of the five types of NPs by the ORG-1 strain were studied and the five models were plotted as 3D surface and 2D contour graphs as follows Fig. 2 for Co_3_O_4_NPs, Fig. 3 for CuONPs, Fig. 4 for Fe_3_O_4_NPs, Fig. 5 for NiONPs, and Fig. 6 for ZnONPs. These graphs represented the recorded responses (NPs concentration) as a function of two parameters at a time while maintaining the other parameter at a fixed level (center point). Fig. 2, Fig. 3, Fig. 4, Fig. 5, Fig. 6 presented different shapes confirming that the presence of variations between the combined effect of three parameters controlling the production of the five types of NPs on the recorded yield of NPs. Table 5 presented the optimum levels of the tested factors necessary to achieve the highest production rates of the five types of NPs by the ORG-1 strain.

**Table 5:** Optimum levels obtained from RSM, predicted, and actuals values for production of Co_3_O_4_NPs, CuONPs, Fe_3_O_4_NPs, NiONPs, and ZnONPs (mg L^−1^) by *A. terreus* ORG-1.

NPs	Optimum levels			NPs concentration (mg L^−1^)	
	Time (min)	Volume of filtrate (L)	Salt concentration (mM)	Actual	Predicted
Co_3_O_4_NPs	150.718	2.687	10.730	338.51 ± 10.44^d^	334.422
CuONPs	371.371	2.324	11.631	335.89 ± 14.65^d^	332.054
Fe_3_O_4_NPs	349.762	2.291	11.748	230.55 ± 11.99^c^	227.790
NiONPs	313.238	3.000	13.730	765.45 ± 21.34^b^	768.728
ZnONPs	265.950	2.017	10.984	862.55 ± 16.52^a^	864.402

SAB broth (pH 6.0) was used for cultivation. Static cultures were carried out for 9 days at 30 °C using an inoculum size of 2 mL/50 mL medium. Calculated mean is for triplicate measurements from two independent experiments ± SD, ^a−f^ means with different superscripts in the same column are considered statistically different (LSD test, *P* ≤ 0.05).

### 3.3 Validation of the five models

To evaluate the performance of parameters controlling the production of the five types of NPs in their optimum levels on the maximization of the recorded yield of NPs by the fungal strain, the five types of NPs were prepared using the optimized levels of parameters listed in Table 5. The obtained results revealed that the predicted values of the NPs’ concentrations by the software (at the optimum levels of the three parameters) were in accordance with the recorded actual concentrations of the five types of NPs. Furthermore, the recorded data (from the two independent experiments) were in a good agreement with the predicted data by the software (Table 5), confirming the high accuracy of the five models used for all types of NPs.

### 3.4 Influence of gamma irradiation on the production of NPs

Table 6 illustrated the effect of exposure of spores of *A. terreus* ORG-1 to gamma-irradiation at various doses on the growth and production of the five types of NPs, as compared with the control (without irradiation). The remarkable features of the recorded data were the significant reduction in the fungal growth after gamma irradiation; meanwhile, the achieved yields of the five types of NPs were significantly increased. The obtained results further revealed that this effect was found to be dose-related and 1 KGy was the best irradiation dose for maximum yields of Co_3_O_4_NPs (545.71 ± 10.19 mg L^−1^), CuONPs (651.67 ± 8.13 mg L^−1^), Fe_3_O_4_NPs (463.19 ± 4.98 mg L^−1^), NiONPs (954.88 ± 13.61 mg L^−1^), and ZnONPs (1356.42 ± 15.18 mg L^−1^). Table 6 also indicated that increasing irradiation doses to 2 or 4 KGy resulted in lower production or no detectable concentrations of NPs. respectively.

**Table 6:** Effect of different gamma irradiation doses on survival rate (%), growth (g L^−1^), and production of Co_3_O_4_NPs, CuONPs, Fe_3_O_4_NPs, NiONPs, and ZnONPs (mg L^−1^) by *A. terreus* ORG-1.

Dose (KGy)	Survival (%)	Dry biomass (g L^−1^)	NPs concentration (mg L^−1^)				
			Co_3_O_4_NPs	CuONPs	Fe_3_O_4_NPs	NiONPs	ZnONPs
0.00 (C)	100	14.65 ± 0.21^a^	335.19 ± 6.87^d^	333.17 ± 7.61^d^	225.85 ± 5.07^d^	765.55 ± 11.53^c^	850.153 ± 10.66^d^
0.250	94.76	12.96 ± 0.56^ab^	417.56 ± 10.72^c^	398.71 ± 7.61^c^	319.56 ± 2.11^c^	863.19 ± 10.56^b^	973.18 ± 12.88^c^
0.500	82.77	10.86 ± 0.43^b^	482.65 ± 9.17^b^	420.77 ± 5.19^b^	371.65 ± 1.87^b^	882.71 ± 9.16^b^	1091.67 ± 11.86^b^
1.00	50.21	8.85 ± 0.18^b^	545.71 ± 10.19^a^	651.67 ± 8.13^a^	463.19 ± 4.98^a^	954.88 ± 13.61^a^	1356.42 ± 15.18^a^
2.00	2.76	1.02 ± 0.15^c^	286.71 ± 4.12^e^	178.65 ± 3.18^e^	85.55 ± 1.32^e^	671.91 ± 10.41^d^	772.66 ± 0.11^e^
4.00	0.00	0.00^d^	0.00^f^	0.00^f^	0.00^f^	0.00^e^	0.00^f^

SAB broth (pH 6.0) was used for cultivation. Static cultures were carried out for 9 days at 30 °C using an inoculum size of 2 mL/50 mL medium. Calculated mean is for triplicate measurements from two independent experiments ± SD, ^a−f^ means with different superscripts in the same column are considered statistically different (LSD test, *P* ≤ 0.05).

## 4 Discussion

Synthesis of nanomaterials using microbial cultures has emerged as a promising biotechnological-based manufacturing process that will aid in the development of more innovative and sustainable industrial nano-manufacturing (Grasso et al., 2021). Moreover, fungi were recently suggested as the most promising bio-factories for the production of nanomaterials (Rao et al., 2017, Mousa et al., 2021) that will certainly bang all doors of the medical, industrial, agricultural sectors (Grasso et al., 2021). Thus, this paper was conducted to explore the possibility of enhancing the production rates of five different types of NPs by the endophytic fungal strain A. terreus ORG-1. Certainly, the standardization of fungal culture growth protocols through studying and optimizing the key culture conditions are potentially crucial for the implementation (Rao et al., 2017), control, (Grasso et al., 2021), and tuning overproduction of nanomaterials by microbes (El-Sayed et al., 2020b). In this study, six types of broth media were evaluated for their effect on production of the five types of NPs by the ORG-1 strain. Among the media tried, SAB broth showed the maximum productivities of Co_3_O_4_NPs, CuONPs, Fe_3_O_4_NPs, NiONPs, and ZnONPs recording 11.53, 25.53, 5.95, 15.85, and 60.73 mg L^−1^, respectively. Similarly, Alam et al. (2017) screened the effects of three different liquid culture media (PD, Czapex Dox, and Yeast malt extract broths) on the synthesis of AgNPs by *Aspergillus niger* and found that Czapex Dox broth was the best one.

Here, the initial yields of the five types of NPs by the ORG-1 strain using PD broth were too low to establish a cost-effective process. Thus, bioprocess optimization protocol was applied primarily via the OFAT method for selecting the most favorable culture conditions suggested to be critical for the production of NPs. The production of the five types of NPs by the ORG-1 strain in this study was significantly enhanced under frmntation conducted by cultivation of 2 mL (10^6^ spore mL^−1^) from a 7-day-old culture in a 50 mL SAB broth (pH 6.0) incubated at 30 °C for 9 days. In our previous study (Mousa et al., 2021), we concluded that synthesis of all the five types of NPs occurred through reduction of metal salts by metabolites produced by the *A. terreus* ORG-1. Similarly, several reports confirmed the role of the fungal secretome including extracellular enzymes (Chatterjee et al., 2020, Omran et al., 2020), proteins, (El-Batal et al., 2020), and bioactive metabolites in both synthesis and stabilization of NPs (Abdelhakim et al., 2020, El-Sayed et al., 2020a, El-Sayed et al., 2020b, El-Sayed et al., 2020c, El-Sayed et al., 2020d, El-Sayed et al., 2020e, El-Sayed et al., 2020f, Feroze et al., 2020, Salem et al., 2021). It is widely known that environmental factors have a key role in the formation of active metabolites of a fungal secretome (McCotter et al., 2016). Culture conditions profoundly modulate the growth and metabolism of fungi and are the critical components, directly affecting the process economics and productivity (Saxena et al., 2016, Fouda et al., 2018, Liang et al., 2019). In agreement with our results, previous reports concluded that the optimum conditions for synthesis of some NPs by different microbal cultures do not always match with the most favorable for growth of this micobe (Ayano et al., 2015, Brooks and Lefebvre, 2017).

In the current study, reaction time (min), volume of fermentation medium (L), and salt concentration were optimized by RSM. These parameters control the process of NPs’ synthesis. The obtained results confirmed that the five models applied for the five types of NPs were significant. In addition, the actual recorded data (yield of NPs) from the validation experiments showed a good agreement in with values predicted by the software, confirming the high accuracy of the five models. In the literature, several reports have investigated the optimization of numerous parameters controlling the biosynthesis of different types of NPs by different fungal strains (Shaheen et al., 2021) and references therein. For example, optimization of rection conditions during synthesis of NPs promoted significantly the output of AgNPs from both *F. oxysporum* (Husseiny et al., 2015) and *Trichoderma viride* (Othman et al., 2017), ZnONPs from *Sclerotinia sclerotiorum* (Fouda et al., 2018), Co_3_O_4_NPs from *A. brasiliensis* (Omran et al., 2020), and AuNPs from *A. terreus* (Balakumaran et al., 2016), and *C. cladosporioides* (Joshi et al., 2017).

In this study, the yield obtained from the five types of NPs was significantly intensified following the exposure of spores of the *A. terreus* to gamma rays at a dose of 1.00 KGy. In agreement with our results, the same irradiation dose was applied to increase the yield of SeNPs (El-Sayed et al., 2020a) and cobalt-ferrite NPs by *Monascus purpurues* (El-Sayed et al., 2020b). In general, exposure to physical mutagens like gamma rays at certain doses may have a stimulatory effect on the enhancement of the fungal secretome responsible for the formation of NPs. Exposure to gamma rays may also induce mutagenesis in the fungal cell by changing the genomic DNA (Zaki et al., 2019). Gamma rays amongst the ionizing radiation are characterized by their strong effects on a living cell that can induce DNA repair mechanisms thereby changing the genes of a living cell (Thacker, 1999, Zaki et al., 2021, Hasanien et al., 2021). Our results showed that gamma-irradiation had a negative effect on growth. Similarly, the fungal biomasses of two mycophenolic acid-producing *P. roqueforti* strains were significantly decreased after exposure of their spores to gamma rays at 0.75 KGy (El-Sayed et al., 2019a). Moreover, lower fungal growth of *A. ochraceus* (an ochratoxin-producing fungus) than control was observed after exposure to gamma rays at a dose of 1.5 KGy (Paster et al., 1985, El-Sayed et al., 2019b, El-Sayed et al., 2019c). In literature, gamma-irradiation is highly recommended for the improvement of microbial strains and production enhancement of a wide array of important metabolites (Zaki et al., 2020, El-Sayed et al., 2020c, El-Sayed et al., 2020d), and references therein. It is widely known that the use of gamma irradiation mutagenesis for the improvement of microbial strains could result in the development of hyper-producing strains with improved production rates that effectively will lower the cost of the production (Parekh et al., 2000, Awan et al., 2011, El-Sayed et al., 2020e, El-Sayed et al., 2020f, Zaki and El-Sayed, 2021).

## 5 Conclusion

In summary, a new bio-factory for the production of Co_3_O_4_NPs, CuONPs, Fe_3_O_4_NPs, NiONPs, and ZnONPs by the endophytic *A. terreus* was successfully developed for the first time. The main fermentation conditions and physical conditions controlling synthesis of these NPs were optimized resulting in an expected increase in the yield of NPs. Furthermore, the achieved yield of these NPs was further intensified using exposure to gamma rays. The final concentrations of the respective NPs reached 545.71, 651.67, 463.19, 954.88, 1356.42 which represents approximately 70, 76, 168, 482, 53.95-fold of their respective initial titers before optimization. Accordingly, these findings greatly recommend the fungal strain as an attractive platform for the synthesis of NPs that can open up the way towards the industrial production of these nanomaterials. Current research work is in progress to scale up the production of the five types of NPs using an industrial scale batch-fermentor.

## Declaration of Competing Interest

The authors declare that they have no known competing financial interests or personal relationships that could have appeared to influence the work reported in this paper.
